# Electronic Nicotine Delivery System Advertisement Trends After US Federal Policy Changes

**DOI:** 10.1001/jamanetworkopen.2024.59188

**Published:** 2025-02-12

**Authors:** Rui Shi, Amal Khayat, Juhan Lee, Kathleen A. Garrison, Rime Jebai, Olivia A. Wackowski, Jenny E. Ozga, Cassandra A. Stanton

**Affiliations:** 1 Department of Communication Studies, Ric Edelman College of Communication & Creative Arts, Rowan University, Glassboro, New Jersey; 2 Braun School of Public Health and Community Medicine, Hadassah Medical Organization, Hebrew University of Jerusalem, Jerusalem, Israel; 3 Department of Epidemiology & Population Health, Albert Einstein College of Medicine, Bronx, New York; 4 Department of Psychiatry, Yale School of Medicine, New Haven, Connecticut; 5 Department of Health Law, Policy & Management, School of Public Health, Boston University, Boston, Massachusetts; 6 Institute for Nicotine and Tobacco Studies, Rutgers University, Brunswick, New Jersey; 7 Behavioral Health & Health Policy, Westat, Rockville, Maryland

## Abstract

**Importance:**

The US has implemented several regulatory measures to govern the sale and marketing of electronic nicotine delivery systems (ENDS). A nicotine warning statement requirement became effective in September 2018, the minimum legal age for tobacco and nicotine sales was raised to 21 years (Tobacco to 21 Act [T21]) in December 2019, and a January 2020 enforcement policy limited the sale of cartridge-based ENDS to only tobacco and menthol flavors.

**Objective:**

To track the presence of the nicotine warning statement, youth appeals, and flavor cues in the advertisements of 3 ENDS brands with marketing authorization during the implementation of the US federal tobacco policies.

**Design, Setting, and Participants:**

In this qualitative study, a content analysis was conducted on ENDS advertisements in the market from November 18, 2015, to June 26, 2022. All mobile, online display, outdoor, and print advertisements from the 3 US ENDS brands with marketing granted orders as of June 2022 were collected through Vivvix, a commercial advertisement intelligence company. Advertisements were excluded if they targeted businesses rather than consumers, contained display errors that were unretrievable, or were duplicates. Data analysis was completed in March 2024.

**Exposure:**

The nicotine warning statement requirement, T21, and the flavor enforcement policy.

**Main Outcomes and Measures:**

Nicotine warning statement compliance and presence of youth-appealing features (eg, young models, sports, and sex appeal) and nonmenthol, nontobacco flavor cues (eg, images of food, characterizing words like *sweet* or *fruity*) in ENDS advertisements before and after policy implementation.

**Results:**

Of the 614 advertisements analyzed, the proportion containing the nicotine warning statement increased from 18 of 83 (21.7%) in the year preceding policy implementation to 147 of 152 (96.7%) in the subsequent year (χ^2^_1_ = 144.5; *P* < .001). The proportion of advertisements containing any youth-appealing features increased from 35 of 171 (20.5%) in the year before T21 to 64 of 143 (44.8%) in the year after T21 (χ^2^_1_ = 21.28; *P* < .001). There was no significant difference in flavor cues in the advertisements in the year before the flavor enforcement policy (30 of 161 [18.6%]) and those in the following year (38 of 149 [25.5%]) (χ^2^_1_ = 2.13; *P* = .14).

**Conclusions and Relevance:**

This qualitative study found immediate compliance with nicotine warning statements but no significant reduction in use of flavor cues and an increase in youth-appealing features in the first year after regulatory measures. The findings suggest a need for continued close monitoring of youth appeals in ENDS advertisements on all platforms to inform potential policies and interventions that can be used to counter the reach, appeal, and impact of these advertisements.

## Key Points

**Question:**

Have advertising strategies of authorized electronic nicotine delivery systems (ENDS) changed within the evolving US federal policy landscape?

**Findings:**

This qualitative study of 614 ENDS advertisements found that while the US policy for nicotine warning statements gained immediate compliance, advertisements showed no change in the use of flavor cues a year after flavor restrictions were enforced. Despite the federal Tobacco to 21 Act, youth-appealing features in e-cigarette advertisements persisted.

**Meaning:**

The findings suggest that close monitoring of flavor cues and youth appeals in ENDS advertisements is essential to inform potential policies and interventions that counter the reach, appeal, and impact of these advertisements.

## Teaser

This qualitative study examines whether changes to US federal policy regarding advertisement of electronic nicotine delivery systems were associated with changes in advertising strategy among brands with marketing authorization.

## Introduction

Electronic nicotine delivery systems (ENDS), also known as e-cigarettes or vapes, are battery-powered devices that heat liquid into aerosol for people to inhale. Most ENDS contain nicotine, which is addictive and has adverse effects on brain development among youths.^1^ ENDS use is also positively associated with mental health problems, such as depression and anxiety, among young people.^2^

Although ENDS entered the US market in 2007,^3^ the US Food and Drug Administration (FDA) Center for Tobacco Products (CTP) did not have the authority to regulate ENDS until 2016, when the Deeming Rule extended FDA authority to all tobacco and nicotine products.^4,5^ Since then, several additional regulatory measures have gone into effect. In September 2018, the FDA enacted a nicotine warning requirement mandating ENDS advertisements to include a warning statement about the addictiveness of nicotine. In December 2019, the US Congress passed the Tobacco to 21 Act (T21), raising the minimum federal legal age for tobacco sales from 18 to 21 years. In January 2020, the FDA issued a flavor enforcement policy that limited the sale of cartridge-based ENDS to only tobacco and menthol flavors (banning all other flavors, such as sweet and fruit flavors, in cartridge-based ENDS). In 2020, the FDA also set a final date (September 9, 2020) by which existing ENDS on the market needed to submit a premarket tobacco product application (PMTA) to the CTP for review to authorize sale and distribution in the US.^4^ As of November 2024, the CTP had authorized the marketing of over 30 tobacco-flavored and 4 menthol-flavored ENDS products and devices,^6^ all from 3 brands (NJOY, Vuse, and Logic).

While these regulations have aimed to discourage youths from using ENDS, it is unknown how ENDS advertising strategies may have changed during this period. Advertising research is important given concerns that exposure to ENDS marketing among youths who have never smoked cigarettes may lead to positive attitudes toward and intentions to use ENDS.^7,8,9,10^ Positive associations have been found between exposure to ENDS advertisements and increased ENDS curiosity, experimentation, intention to use, and use among youths^11,12,13,14,15,16,17^ as well as higher susceptibility to other tobacco product use (eg, cigarettes).^11,14,18^

To appeal to youths, ENDS advertisements use tactics similar to those used for traditional cigarette advertising, such as associating ENDS with independence, rebellion, and social acceptance.^8^ ENDS advertisements also emphasize themes such as the “cool” factor, modernity, social status, romance, celebrity endorsements, and technology.^8,19,20,21,22^ Other common youth-appealing strategies include promoting fruity and sweet flavors, price promotions, harm reduction claims, sports events sponsorships, emotional appeals, and humor.^9^ However, exposure to health warnings in ENDS advertisements may reduce product curiosity and intention to use.^10,23^ Continued monitoring of ENDS advertising is therefore important to inform regulation of ENDS marketing practices, especially for brands that have received FDA marketing authorization. The goal of this study was to examine changes in ENDS advertising strategies for brands with CTP premarket authorization within the evolving US federal policy landscape.

## Methods

This qualitative study involved a content analysis of ENDS advertisements to examine advertising strategies and any change in strategies related to US federal policy changes. The Rowan University institutional review board exempted the study from approval as no human participants were involved. The Standards for Reporting Qualitative Research (SRQR) reporting guideline was followed.

### Data Collection and Sample

Advertisements (mobile or online, outdoor, and print) from Vuse, NJOY, and Logic brand ENDS across national and regional media markets in the US were collected through Vivvix, a commercial advertisement intelligence company.^24^ These brands were selected because they were the only ones with at least 1 device with a marketing granted order (MGO) from the CTP as of the study onset in June 2022. Advertisements in the sample were run in the consumer market from November 18, 2015, to June 26, 2022. We excluded advertisements that targeted businesses rather than consumers (eg, *Convenience Store Products* magazine advertisements), advertisements with display errors that were unretrievable, or duplicates.

### Content Analysis

A codebook was abductively developed based on previous research on ENDS advertisements and decision summaries of the CTP for MGOs,^6^ which named some youth-appealing content features to avoid in product marketing and provided recommendations for ENDS advertising practices. An inductive approach was used to review sample advertisements and suggest additional items for the codebook. The final coding scheme is shown in Table 1. Advertisements were coded by 2 research assistants (Cohen κ, 0.66-1.00) on features of the nicotine warning statement,^25^ youth appeal (eg, featuring models <25 years of age; depicting sex, sports, or celebrity^8,20,26^), and flavor cues (eg, showing images of food items; using words like *sweet*, *fruity*, *candy*, *sugary*, or *gummy*^21^).

**Table 1.** Content Coding Items Across 3 Electronic Nicotine Delivery System Brands With Premarket FDA Authorization as of August 2022^a^

Variable	Ads, No. (%)				*P* value^b^
	All (N = 614)	Vuse (n = 483)	NJOY (n = 56)	Logic (n = 75)	
Ad type					
Mobile or online	510 (83.1)	403 (83.4)	50 (89.3)	57 (76.0)	<.001
Outdoor	63 (10.3)	39 (8.1)^c^	6 (10.7)^c^	18 (24.0)^c^	
Print	41 (6.7)	41 (8.5)	0	0	
Nicotine warning statement					
None	73 (11.9)	14 (2.9)	53 (94.6)^c^	6 (8.0)	<.001
<1/8 of Ad	85 (13.8)	83 (17.2)^c^	1 (1.8)	1 (1.3)	
>1/8 of Ad	456 (74.3)	386 (79.9)	2 (3.6)^c^	68 (90.7)	
Congregated youth appeals					
Overall	199 (32.4)	164 (34.0)	9 (16.1)^c^	26 (34.7)	.02
Model age, No./total No. (%)					
19-25 y	14/86 (16.3)	12/63 (19.0)	1/8 (12.5)	1/15 (6.7)	.01^d^
30-40 y	36/86 (41.9)	32/63 (50.8)	1/8 (12.5)	3/15 (20.0)	
>45 y	4/86 (4.7)	3/63 (4.8)	0	1/15 (6.7)	
Could not be determined	32/86 (37.2)	16/63 (25.4)	6/8 (75.0)	10/15 (66.7)	
Cartoon	1 (0.2)	0	0	1 (1.3)	.21^d^
Celebrity	0	0	0	0	NA
Testimonial	0	0	0	0	NA
Sports	22 (3.6)	22 (4.6)	0	0	.04^d^
Music	20 (3.3)	20 (4.1)	0	0	.08^d^
Success	9 (1.5)	3 (0.6)^c^	2 (3.6)^c^	4 (5.3)^c^	.004^d^
Sex	3 (0.5)	3 (0.6)	0	0	>.99^d^
Advanced technology	73 (11.9)	50 (10.4)	4 (7.1)	19 (25.3)^c^	<.001
Product personalization	56 (9.1)	56 (11.6)^c^	0	0	<.001
Environmentally friendly	22 (3.6)	17 (3.5)	2 (3.6)	3 (4.0)	.92^d^
Sociability	24 (3.9)	19 (3.9)	1 (1.8)	4 (5.3)	.60^d^
Freedom or choice	30 (4.9)	28 (5.8)	0	2 (2.7)	.11^d^
Congregated flavor cues	135 (22.0)	107 (22.2)	11 (19.6)	17 (22.7)	.90
Food image	9 (1.5)	2 (0.4)	7 (12.5)^c^	0	<.001^d^
Food words	38 (6.2)	32 (6.6)	0	6 (8.0)	.07^d^
Nontobacco or nonmenthol flavor words	159 (25.9)	129 (26.7)	4 (7.1)^c^	26 (34.7)	.001
Sweetness words	33 (5.4)	27 (5.6)	0	6 (8.0)	.09^d^
Other content coding items					
Mention of menthol taste	39 (6.3)	30 (6.2)^c^	0^c^	9 (12.0)^c^	.01^d^
Usability features	100 (16.3)	71 (14.7)	4 (7.1)	25 (33.3)^c^	<.001
Emphasis on good experience	30 (4.9)	22 (4.5)	6 (10.7)	2 (2.7)	.10^d^
Color scheme					
Black and white	63 (10.2)	60 (12.4)^c^	3 (5.4)^c^	0^c^	<.001^d^
1-3 Colors	517 (84.2)	391 (81.0)^c^	53 (94.6)	74 (98.7)	
4-6 Colors	34 (5.5)	0	1 (1.8)	1 (1.3)	
Made in US	11 (1.8)	11 (2.3)	0	0	.37^d^
Price promotion	285 (46.3)	218 (45.1)	32 (57.1)	35 (46.7)	.23
Showing brand website	514 (83.6)	400 (82.8)	50 (89.3)	64 (85.3)	.41
Showing brand social media	1 (0.2)	1 (0.2)	0	0	>.99^d^
Showing product image					
Overall	532 (86.5)	419 (86.7)	46 (82.1)	67 (89.3)	.49
Cigalike	95 (15.4)	59 (12.2)^c^	19 (33.9)	17 (22.7)	<.001
Vape pen	170 (27.6)	90 (18.6)^c^	27 (48.2)^c^	53 (70.7)^c^	<.001
Box mod or tank	2 (0.3)	2 (0.4)	0	0	>.99^d^
Vape pod	317 (51.5)	315 (65.2)	2 (3.6)	0	<.001
Targeting people who smoke cigarettes	33 (5.4)	6 (1.2)^c^	18 (32.1)^c^	9 (12.0)^c^	<.001^d^
Smoking alternative claim	37 (6.0)	9 (1.9)	27 (48.2)^c^	1 (1.3)	<.001^d^
Includes nicotine concentration level	19 (3.1)	18 (3.7)	0	1 (1.3)	.29^d^

Abbreviations: Ad, advertisement; FDA, US Food and Drug Administration; NA, not applicable.

^a^ The first-run dates were March 24, 2016, to March 28, 2022, for Vuse; November 18, 2015, to August 26, 2019, for NJOY; and December 7, 2015, to March 1, 2021, for Logic.

^b^ χ^2^ Test unless otherwise indicated.

^c^ Brand was significantly different from the other 2 brands in Fisher exact pairwise comparisons with Bonferroni correction.

^d^ Fisher exact test.

Coding items were combined into 3 measures relevant to policy responses (Table 1). Violation of the nicotine warning statement requirements was measured as a dichotomous variable with 0 for policy compliance or 1 for violation. Youth appeal was measured as a dichotomous variable that was transformed from 13 content coding items related to youth appeals other than flavor (model age, cartoon, celebrity, testimonial, sports, music, success, sex, advanced technology, product personalization, environmental friendliness, sociability, and freedom or choice), with 0 indicating none of the 13 items and 1 indicating any of the 13 items. Additional analyses were performed on 6 of the 13 youth-appealing features (models aged 19-25 years, celebrity, testimonial, sports, success, and sex) (Table 2) that were explicitly named in the MGO for Vuse. Response to the flavor enforcement policy was measured as a dichotomous variable that was transformed from 5 content coding items related to flavor cues, with 0 indicating no flavor cues and 1 indicating any of the 5 flavor cues. Menthol was coded separately as it was not included in the flavor enforcement policy.

**Table 2.** Presence of Youth-Appealing Features Specified in FDA CTP Marketing Granted Orders Before and After Premarket Authorization for Vuse Solo in October 2021^a^

Feature	Vuse advertisements, No. (%)			
	All (N = 483)	Before PMTA submission (n = 365)	During PMTA pending period (n = 103)	After PMTA authorization (n = 15)
Models aged 19-25 y	12 (2.5)	7 (1.9)	5 (4.9)	0
Celebrity	0	0	0	0
Testimonial	0	0	0	0
Sports	22 (4.6)	12 (3.3)	10 (9.7)	0
Success	3 (0.6)	2 (0.5)	0	1 (6.7)
Sex	3 (0.6)	2 (0.5)	1 (1.0)	0
Any of the above	37 (7.7)	23 (6.3)	13 (12.6)	1 (6.7)

Abbreviations: CTP, Center for Tobacco Products; FDA, US Food and Drug Administration; PMTA, premarket tobacco product application.

^a^ The first-run dates were March 2016 to August 2020 before PMTA submission, September 2020 to October 2021 during the PMTA pending period, and November 2021 to March 2022 after PMTA authorization.

### Statistical Analysis

Content coding results were merged with time stamp data on the date the advertisements entered the media markets. Descriptive statistics were used to examine trends in advertising strategies over time. Coding items were compared between the 3 brands using the χ^2^ test and Fisher exact test^27^ with Bonferroni correction. Additional χ^2^ tests were conducted to compare policy compliance before and after each policy implementation date. Data analysis was completed in March 2024 using SPSS, version 29 (IBM Corp). Two-sided *P* <.05 was considered significant.

## Results

A total of 692 ENDS advertisements were identified. After we excluded those directed toward businesses rather than consumers (n = 72), those with display errors that were unretrievable (n = 5), and duplicates (n = 1), the final analytic sample consisted of 614 ENDS advertisements. Most were placed online in the form of mobile or online display advertisements (510 [83.1%]) followed by outdoor (63 [10.3%]) and print (41 [6.7%]). The majority of the mobile and online display advertisements (462 [90.6%]) guided viewers to brand websites with hyperlinks, link buttons, or QR codes. Brand websites were also frequently linked in print (32 [78.0%]) and outdoor (20 [31.7%]) advertisements.

Nicotine concentrations were shown in 19 advertisements (3.1%). Of those, all but 1 (94.7%) showed the percentage of nicotine per total weight, and nicotine levels in the advertisements ranged from 0.1% to 5.0%, with 1.5%, 2.4%, and 5.0% being the most frequently shown nicotine levels. One advertisement showed nicotine per milliliter of liquid (ie, 18 mg/mL).

### Brand Advertising Strategy Comparison

The sample contained 483 advertisements from Vuse (78.7%; first-run date, March 24, 2016, to March 28, 2022), 56 from NJOY (9.1%; first-run date, November 18, 2015, to August 26, 2019), and 75 from Logic (12.2%; first-run date, December 7, 2015, to March 1, 2021) (Table 1). Vuse advertisements mostly featured vape pods (315 [65.2%]). More Vuse advertisements (56 [11.6%]) featured product personalization (eg, allowing customization or choice of shell colors and patterns) than Logic and NJOY advertisements (both 0; *P* < .001 for both). A nicotine warning statement was present on 469 Vuse advertisements (97.1%), although it was smaller than required in 83 (17.2%). A nicotine warning statement was also present on most Logic advertisements (69 [92.0%]), in contrast to NJOY (3 [5.4%]).

NJOY advertisements mostly featured cigalikes (19 [33.9%]) and vape pens (27 [48.2%]) and used food images (7 [12.5%]) more often than Vuse (2 [0.4%]) and Logic (0) advertisements (*P* < .001 for both). However, NJOY advertisements used nontobacco or nonmenthol flavor words less often (4 [7.1%] vs 129 [26.7%] for Vuse and 26 [34.7%] for Logic; *P* = .001). NJOY advertisements more frequently targeted people who smoke cigarettes by using terms like *smokers* or *if you smoke* or by showing images of cigarettes (18 [32.1%] vs 6 [1.2%] for Vuse or 9 [12.0%] for Logic; *P* < .001). Additionally, NJOY was more likely than the other 2 brands to present ENDS as a cigarette alternative by using words such as *alternative* or *switching* (27 [48.2%] vs 9 [1.9%] for Vuse and 1 [1.3%] for Logic; *P* < .001). No NJOY advertisements were identified after 2019, and all but 3 NJOY advertisements (53 [94.6%]) were run before the required nicotine warning statement was implemented. Consequently, only 3 NJOY advertisements (5.4%) contained a nicotine warning statement.

Most of the Logic advertisements featured vape pens (53 [70.7%]). Compared with Vuse (30 [6.2%]) and NJOY (0), more Logic advertisements mentioned menthol flavor (9 [12.0%]) (*P* = .01). Logic advertisements also focused more on advanced technology (19 [25.3%] vs 50 [10.4%] for Vuse and 4 [7.1%] for NJOY; *P* < .001) and practical usability features, such as no spill, fast charging, or easy to use (25 [33.3%] vs 71 [14.7%] for Vuse and 4 [7.1%] for NJOY; *P* < .001).

### Trends in Nicotine Warning Statements

**Figure 1.**
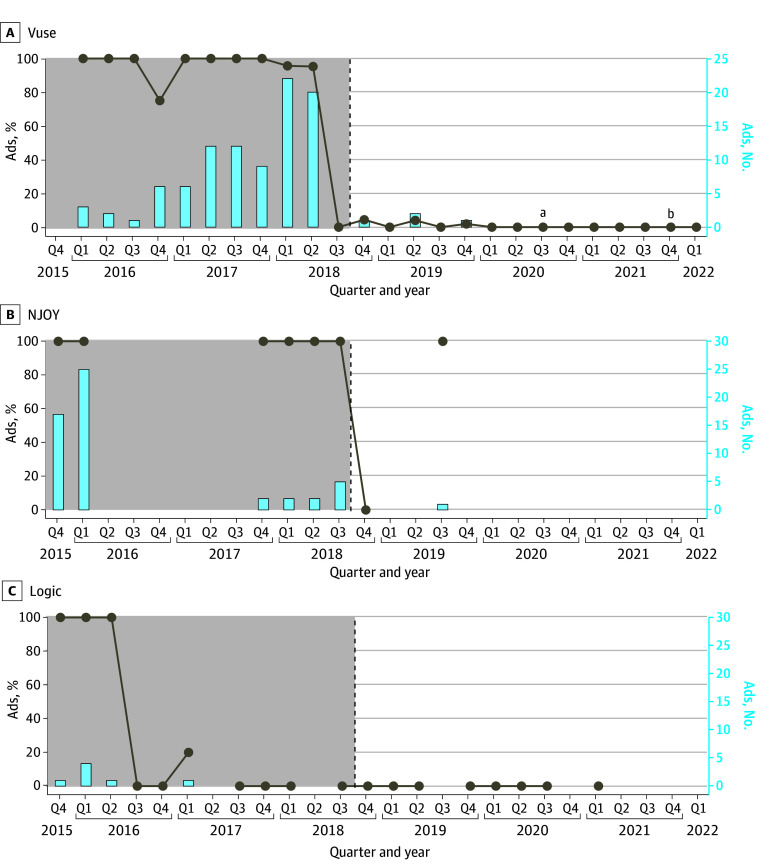
Percentage and Number of Advertisements Violating the Nicotine Warning Statement Requirement Implemented in September 2018 Violation was coded as not showing the nicotine warning statement at all or including a statement smaller than one-eighth of the advertisement. Line breaks indicate quarters for which no advertisements were collected; vertical dashed lines, implementation of nicotine warning statement requirement. ^a^Premarket tobacco product application submission. ^b^Premarket tobacco product application authorization.

The US federal policy requiring a nicotine warning statement was met with instant compliance in all 3 brands (Figure 1). The proportion of advertisements containing the required nicotine warning statement increased from 18 of 83 (21.7%) in the year preceding policy implementation to 147 of 152 (96.7%) in the subsequent year (n = 235; χ^2^_1_ = 144.5; *P* < .001).

For Vuse, 55 of 61 advertisements (90.2%) were noncompliant with the policy 1 year before its implementation compared with 3 of 117 advertisements (2.6%) found in violation 1 year after. For NJOY, all 10 advertisements (100%) were noncompliant 1 year before the policy, with 2 of 4 advertisements (50.0%) noncompliant 1 year after. Logic showed full compliance both the year before (12 advertisements) and the year after (31 advertisements) the policy.

### Trends in Youth Appeals

**Figure 2.**
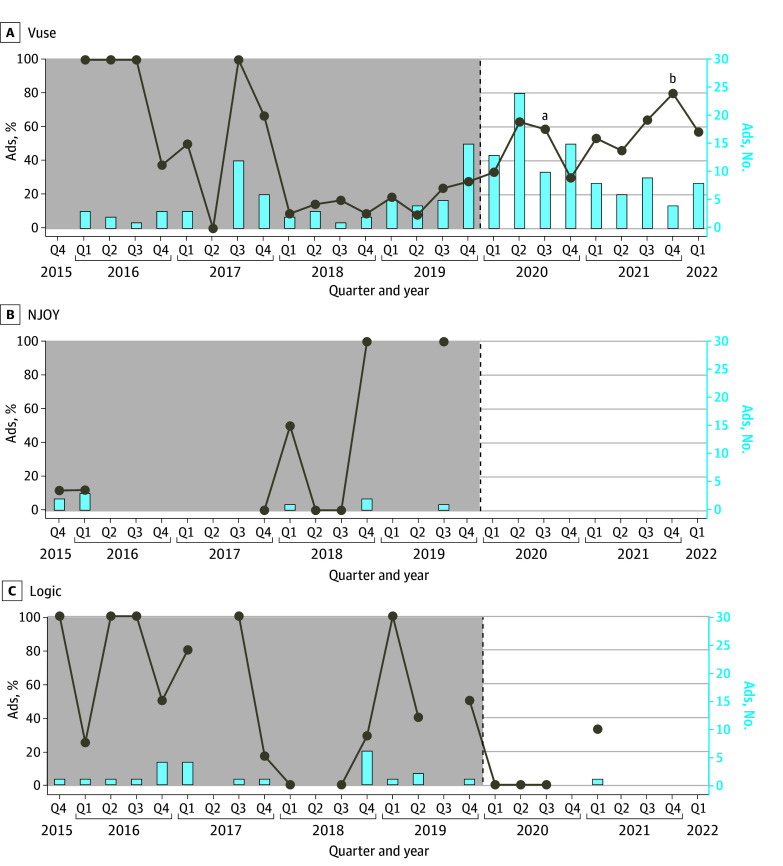
Percentage and Number of Advertisements Containing at Least 1 Youth Appeal Feature Before and After Implementation of Federal Tobacco to 21 Act (T21) in December 2019 Youth appeal features included a model aged younger than 25 years, cartoon, sports, music, success, sex, advanced technology, product personalization, environmental friendliness, sociability, and freedom or choice. Line breaks indicate quarters for which no advertisements were collected; vertical dashed lines, implementation of T21. ^a^Premarket tobacco product application submission. ^b^Premarket tobacco product application authorization.

Youth appeal in ENDS advertisements was prevalent after the date of T21 implementation (Figure 2). The proportion of advertisements containing at least 1 youth-appealing feature increased from 35 of 171 (20.5%) in the year before T21 to 64 of 143 (44.8%) in the year after (n = 314; χ^2^_1_ = 21.28; *P* < .001).

Vuse advertisements showed the greatest increase in youth appeals. The most common youth appeals identified in Vuse advertisements were product personalization (56 [11.6%]), advanced technology (50 [10.4%]), and models who appeared to be 19 to 25 years of age (12 of 63 Vuse advertisements that included human models [19.0%]). Although advanced technology was also a theme observed for Logic (19 advertisements [25.3%]), it was not found in Logic advertisements run after T21.

In the MGO for Vuse, CTP named specific youth-appealing features to avoid in product marketing. We tracked these features along the timeline for PMTA submission and authorization for Vuse. Vuse received its first MGO on December 10, 2021, for Vuse Solo. Table 2 shows the proportion of Vuse advertisements that included features specified in the MGO for Vuse Solo and entered the consumer market before PMTA submission (23 of 365 [6.3%]), during the PMTA pending period (13 of 103 [12.6%]), and after Vuse Solo received authorization (1 of 15 [6.7%)].

### Trends in Flavor Cues

**Figure 3.**
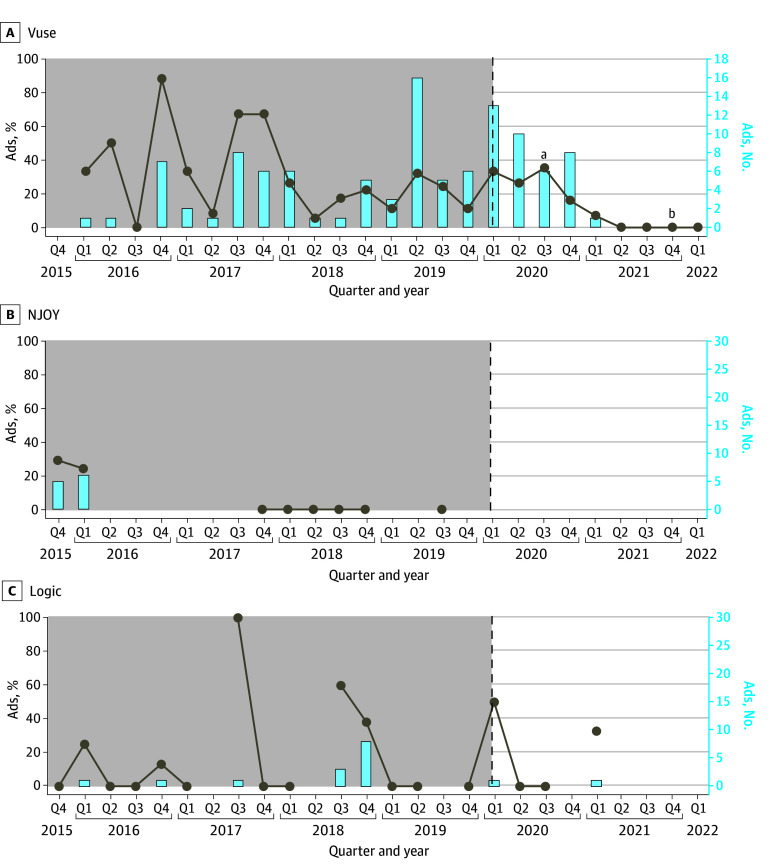
Percentage and Number of Advertisements Containing at Least 1 Nontobacco, Nonmenthol Flavor Cue Before and After Flavor Enhancement Policy Implementation in January 2020 Flavor cues included food image, food text, nontobacco or nonmenthol flavor words, and sweetness words. Line breaks indicate quarters for which no advertisements were collected; vertical dashed lines, implementation of flavor enhancement policy. ^a^Premarket tobacco product application submission. ^b^Premarket tobacco product application authorization.

No change was observed in the prevalence of flavor cues in ENDS advertisements between the year before the flavor enforcement policy (30 of 161 [18.6%]) and the following year (38 of 149 [25.5%]) (n = 310; χ^2^_1_ = 2.13; *P* = .14). It took about 1.5 years for Vuse advertisements to no longer contain flavor cues after the flavor enforcement policy was implemented (Figure 3). The prevalence of menthol cues was 7 of 161 advertisements (4.3%) the year before the flavor enforcement policy and 14 of 149 (9.4%) in the subsequent year (n = 310; χ^2^_1_ = 3.12; *P* = .08).

## Discussion

This qualitative study using a content analysis identified advertising features for the 3 ENDS brands that have received marketing authorization and tracked their changes during the implementation of US federal tobacco policies (nicotine warning statement, T21, flavor enforcement policy, and product-specific marketing authorizations). Advertisements were found to generally comply with FDA nicotine warning statement requirements yet were still found to use youth-appealing features and flavor cues despite relevant policy changes.

Youth appeals were common in ENDS advertisements prior to 2020 (the first year of T21 and the year that PMTAs were due). Vuse advertisements had the highest prevalence of youth-appealing features, particularly related to advanced technology, product personalization, and the use of young models. Highlighting innovative technology is a common marketing practice in ENDS marketing on social media.^28^ Innovative technology in ENDS marketing commonly features newer designs, delivery of stronger nicotine, longer battery time, low odor and vapor, and features such as stealth mode (eg, no vapor, no light) that might be appealing to youths who want to use ENDS more discreetly.^29^ Perceiving ENDS devices as innovative might be related to the adoption of ENDS use among youths.^30^

Personalization was also a common advertising theme, particularly for Vuse. Data suggest that once a person purchases an ENDS device (unless disposable), they will continue using the same device, and these devices might be considered lifestyle accessories among vape communities.^31,32^ More broadly, personalization of products is in demand, in particular from younger customers.^33^ Personalization of ENDS features was reported by 41% of adolescents who currently used ENDS and was named as one of the most appealing product features by adolescents who had never used ENDS.^34^ Therefore, featuring personalization in ENDS advertisements may increase their appeal, particularly to youths. Taken together, the prevalence of youth-appealing features in Vuse advertisements in particular is noteworthy given that data from the US National Youth Tobacco Survey show that Vuse has been and is currently (as of 2024) one of the most popular e-cigarette brands among youths who have used e-cigarettes for several years.^35,36,37^

Similarly to youth appeals after T21, flavor cues in Vuse advertisements did not disappear immediately after flavor restrictions were enforced. Instead, these cues only ceased to appear following the brand’s PMTA submission, roughly 1.5 years after the flavor restrictions were implemented. This trend highlights potential gaps in existing policies and underscores the need for stricter oversight and enforcement to address the use of flavor mentions in ENDS advertising. The issue of flavor restrictions has been contentious for ENDS products given that flavors are appealing to adults who smoke cigarettes (who may potentially benefit from switching to ENDS) and to youths. Of note, the FDA has been conservative to date in terms of authorizing flavored ENDS, with a small initial group of menthol-flavored products only recently authorized.^38^ Continuous surveillance and monitoring of marketing of such flavored products is warranted.

This study also found that ENDS advertisements included the required nicotine warning statement in the year following implementation of this policy in 2018. Exposure to nicotine warnings among youths has been associated with higher perceptions of ENDS risk and addiction,^39,40^ lower use susceptibility,^41^ and greater knowledge that ENDS contain nicotine.^39^ However, as scientific knowledge about potential ENDS risks has expanded, research suggests that additional warnings about other ENDS-related health risks may be beneficial to consumers, including youths.^42,43,44^

Few ENDS advertisements were found to label nicotine concentration levels, and those that did used a variety of metrics (ie, percentage, milligrams per milliliter), making the concentration challenging to interpret. Two studies among youths^45^ and adults^46^ using ENDS found that both groups were unable to correctly distinguish nicotine strength from presented concentration pairs (eg, 24 mg/mL vs 3%) and to underestimate nicotine strength based on these metrics.^46,47^ The authors of these studies^45,46,47^ called for a single, easy-to-understand nicotine labeling system to increase understanding of nicotine content in ENDS products.

Among the 4 policies implemented during the study period, the content of ENDS advertisements, particularly the ones by Vuse, appeared to respond more strongly to the required nicotine warning statement policy and PMTA submission and less to the flavor enforcement policy and T21. This is perhaps not surprising given that T21 and the flavor enforcement policy did not directly address advertisements. We found that Vuse advertisements featuring youth appeals increased after the enactment of T21. To gain PMTA authorization, brands may have been motivated to demonstrate compliance with previous policies and efforts to reduce potential youth appeals, leading to fewer advertisements with youth-appealing features and fewer advertisements overall. For example, in their PMTA applications, Vuse proposed specific advertising features that it would avoid to mitigate youth appeal and use (eg, not to include individuals aged <25 years as models^48^), which were supported and encouraged by the CTP in the MGO. The observed decrease in youth-appealing features in Vuse advertisements in conjunction with the PMTA timeline may suggest benefits of the PMTA process. While mandated advertising restrictions are challenging in the US based on First Amendment grounds, the FDA could recommend that companies expand restrictions to include features like personalization and technology to reduce youth appeal in ENDS advertisements when issuing MGOs. Continued monitoring of youth product use is also critical, as the FDA has the authority to rescind product authorizations if public health risks rise.

### Limitations

This study has some limitations. The sample was limited to static images of ENDS advertisements (ie, not television or radio); thus, dynamic visual and auditory elements that may influence advertisement appeal were not analyzed. The study also focused on paid media advertisements rather than the entire spectrum of ENDS promotional content, such as advertising on brands’ social media or earned media.^49^ No NJOY advertisements were collected after August 2019, and no Logic advertisements were collected after March 2021. It is plausible that these dates mark when both brands ceased advertising in traditional outlets, as they align with the notable decline in ENDS marketing expenditures observed from the fourth quarter of 2019 through the third quarter of 2021.^50^ Although Vivvix did not observe any NJOY advertisements in monitored outlets after 2019, NJOY remains one of the most popular e-cigarette brands (third, following JUUL and Vuse), suggesting that NJOY is advertising in outlets not captured by the present dataset.^51^ Additionally, while to our knowledge, there is no strong evidence regarding the direct impact of the technology and personalization themes on youth ENDS use, data from focus groups have indicated that high-tech elements in advertisements do attract youths’ attention.^22^ Future research should test the effects of these themes in ENDS advertising.

## Conclusions

This qualitative study of ENDS advertisements from 3 brands with FDA authorization found immediate compliance with the US policy for nicotine warning statements after its implementation. In contrast, ENDS advertisements showed no change in the use of flavor cues in the first year after product flavor restrictions and a gradual reduction thereafter, with the most noticeable reductions occurring after PMTA submissions. Despite the federal T21 policy, the use of youth-appealing features in Vuse ENDS advertisements persisted but with some reductions after PMTA submissions and authorization. Exposure to ENDS advertisements is a well-known risk factor for ENDS use among youths.^11,12,13,14,15,16,17^ This study’s findings suggest a need for continued close monitoring of youth appeals in ENDS advertisements on all platforms to inform potential policies and interventions that can be used to counter the reach, appeal, and impact of the advertisements.

## Notes

Supplement.
Data Sharing Statement
