# Supplementary material for: Electronic Nicotine Delivery System Advertisement Trends After US Federal Policy Changes
Source: JAMA Netw Open. 2025 Feb 12;8(2):e2459188. doi: 10.1001/jamanetworkopen.2024.59188 (PMC11822544; doi:10.1001/jamanetworkopen.2024.59188)
Supplement: Supplement. — Data Sharing Statement [file jamanetwopen-e2459188-s001.pdf]

Data Sharing Statement

Shi. Electronic Nicotine Delivery System Advertisement Trends After US Federal Policy Changes. *JAMA Netw Open*. Published February 12, 2025.
doi:10.1001/jamanetworkopen.2024.59188

Data
Data available: Yes
Data types: Data (not involving human participants), Data dictionary
How to access data: Available upon reasonable request from corresponding author at shi@rowan.edu
When available: With publication

Supporting Documents
Document types: None

Additional Information
Who can access the data: researchers whose proposed use of the data has been approved
Types of analyses: none
Mechanisms of data availability: with investigator support
Any additional restrictions: The author team will not share the actual ads due to contract restrictions with Vivvix. The codebook for this content analysis and the coding results of the ads are available upon reasonable request.
